# A review on Sero diversity and antimicrobial resistance patterns of *Shigella* species in Africa, Asia and South America, 2001–2014

**DOI:** 10.1186/s13104-016-2236-7

**Published:** 2016-08-30

**Authors:** Atsebaha Gebrekidan Kahsay, Saravanan Muthupandian

**Affiliations:** Department of Medical Microbiology and Immunology, Institute of Biomedical Sciences, College of Health Sciences, Mekelle University, Mekelle, Tigray Ethiopia

**Keywords:** Serogroups of *Shigella*, Antimicrobial resistance

## Abstract

**Background:**

*Shigella*, gram negative bacterium, is responsible for Shigellosis/bacillary dysentery. It is a global concern although it predominates in developing countries. These are *Shigella dysenteriae, Shigella flexneri, Shigella boydii* and *Shigella sonnei.* Drug resistance by *Shigella* species is another headache of the world. Therefore; this study aimed to review distribution of *Shigella* Serogroups and their antimicrobial patterns carried out in Africa, Asia and South America.

**Methods:**

A literature search was performed to identify published studies between January 2001 and December 2014. Published studies were identified using an initial search of the MEDLINE/Index Medicus Database, PubMed, Project Management Consultant, Google Scholar, Science Direct, BioMed Central and Index Copernicus.

**Results:**

*Shigella flexneri* was isolated predominately from seven studies in four African countries and eight studies in five Asian countries. The countries in which eligible studies carried out were Ethiopia, Kenya, Eritrea and Ghana in Africa and Pakistan, Iran, China, Nepal and India in Asia. *S. sonnei* was isolated predominately from one study in Africa, four in Asia and two South America. The countries in which eligible studies carried out were Ethiopia from Africa, Thailand, Vietnam and Iran from Asia and Chile and Trinidad from South America. *S. dysentery* was also reported majorly from one eligible study in Egypt and one in Nepal. *S. boydii* did not score highest prevalence in any one of the eligible studies. Three studies from Africa, five from Asia and one from South America were reviewed for antimicrobial resistance patterns of *Shigella* Serogroups. In all the regions, Ampicillin developed highly resistance to almost all the Serogroups of *Shigella* whereas all the strains were sensitive to Ciprofloxacin.

**Conclusion:**

The incidence of *Shigella* Serogroups in the selected three regions is different. The domination of *S. flexneri* is observed in Africa and Asia although *S. sonnei* in South America is dominant. *Shigella* Serogroups are becoming resistance to the commonly prescribed antimicrobial drugs in developing countries.

## Background

*Shigella*, a group of Gram-negative, non-spore forming and rod shaped bacterium, is the causative agent of shigellosis (or bacillary dysentery). *Shigella* Serogroups are considered to be highly infectious due to their low infectious dose (10–100 organisms) [1]. *Shigella* is primarily transmitted through the fecal-oral route; therefore, it is still a main global public health threat, particularly in developing countries due to poor sanitation conditions [2].

*Shigella* bacteria are serologically grouped into four species named as *Shigella dysenteriae, Shigella flexneri, Shigella boydii* and *Shigella sonnei*. However Serogroups of *Shigella* bacteria have similar property of pathogenesis and epidemiologically they have peculiar characteristics. *S. flexneri* found predominantly in developing world, while *S. sonnei* is the most common species found in the industrialized countries [3]. The severity of Serogroups of *Shigella* is different one from the other that *S. sonnei* and *S. flexneri* cause mild infection whereas *S. boydii* and *S. dysenteriae* cause severe and most serious infection respectively [2]. The infection caused by *S. sonnei* and *S. boydii* lasts with short duration and mostly found in industrialized countries. The distribution of *Shigella sonnei* in the United States of America is 74–79 % [4] and 61 % in Europe [5].

The emerging of multi drug resistance is becoming a serious problem in the treatment of shigellosis. An increment of multidrug resistance to shigellosis is equivalent to a widespread uncontrolled use of antibiotics in developing countries. This emergency of drug resistance calls for the rational use of effective drugs and underscores the need for alternative drugs to treat infections caused by resistant strains [6].

*Shigella* is more associated with low socio economic status and poor sanitation of under developed countries [2]. Researches were done by different researchers concerning the Serogroups and antimicrobial susceptibility patterns of *Shigella* in Africa, Asia and South America as a result this study aimed in reviewing the distribution of Serogroups of *Shigella* and the resistance patterns of antimicrobial drugs which were conducted in the developing countries in the past 14 years.

## Methods

### Search strategy and selection criteria

A literature search was performed to identify published studies between January 2001 and December 2014. Published studies were identified using an initial search of the MEDLINE/Index Medicus Database, PubMed, PMC, Google Scholar, Science Direct, BioMed Central and Index Copernicus.

The study initially screened all unique publications for eligibility based on the relevancy of the title and then screened the full manuscripts for inclusion and exclusion criteria. The following Keywords were used to search all the published papers from the above engines. These were Prevalence, isolation, Sero diversity, occurrence, epidemiology, Magnitude, burden, estimation, distribution, diversity and antimicrobial resistance patterns of *Shigella.* Studies conducted before 2001 and researches which concerned only prevalence and antimicrobial susceptibility patterns of *Shigella* were excluded.

### Data extraction

The first author, country, year of publication, setting, sample size, children and all age, sample source, prevalence and distribution of Serogroups of *Shigella* were extracted from the eligible studies. Ampicillin, Tetracycline, Chloramphenicol, Ciprofloxacin, Cotrimoxazole, Nalidixic acid and Gentamicin resistance patterns of the four Sero groups of *Shigella* were extracted from the eligible studies.

Studies were included in the review if they fulfill the following criteria:

- Isolation and identifying of *Shigella* from stool samples should be based on standard bacteriological methods and *Shigella* Serogroups were detected serologically using slide agglutination and antimicrobial resistance patterns of *Shigella* Serogroups should be based on Clinical Standards Laboratory Institute guidelines using disc diffusion methods.
- Full text articles studied in Africa, Asia, and South America and published in English everywhere in the globe were included.

## Results

Majority of the reviewed articles were Hospital based studies. About 40 % of the study participants were children under 15 years old. Stool samples were the source of specimens in all the eligible studies. A total of 69,849 stool specimens’ data were collected. Eighteen (72 %) of the eligible studies were published before 2010 and the rest seven were published from 2010 to 2014 (Table 1).

**Table 1:** Characteristics of appropriate studies and distribution of Serogroups of *Shigella* reviewed from Africa, Asia and South America from 2001 to 2014

First author	Country	Pub. year	Setting	Age group	Sample sources	Sample size	Shig. Pos. (N)	*Shigella* Serogroups Pos. N (%)			
								A	B	C	D
Mache [8]	Ethiopia	2001	Hosp & HC	Children	Stool	384	77	23 (29.9)	31 (40.3)	15 (19.5)	8 (10.4)
Brooks [9]	Kenya	2003	Laboratory	All ages	Stool	2374	198	80 (40.4)	97 (49)	13 (6.6)	8 (4)
Abu-Elyazeed [10]	Egypt	2004	Surveillance	Children	Stool	696	131	74 (56.5)	30 (22.9)	26 (19.8)	1 (0.8)
Chompook [18]	Thailand	2005	Population	All ages	Stool	6536	146	–	24 (16.4)	–	122 (83.6)
Wang [19]	China	2005	Community	All ages	Stool	10,105	331	–	306 (93)	–	25 (7)
Bhattacharya [20]	Nepal	2005	Hospital	All ages	Stool	1396	53	39 (73.6)	12 (22.6)	2 (3.8)	–
Fulla [21]	Chile	2005	HC	Children	Stool	4080	178	–	77 (43.3)	–	101 (56.7)
Mashouf [22]	Iran	2006	Hospital	Children	Stool	1686	166	56 (33.7)	67 (40.3)	25 (15)	18 (11)
Wilson [23]	Nepal	2006	Hospital	All ages	Stool	770	83	12 (14.5)	56 (67.5)	5 (6.0)	10 (12)
Nguyen [24]	Vietnam	2006	Hospital	All ages	Stool	587	28	–	7 (25)	1 (3.6)	20 (71.4)
Naik [11]	Eritrea	2006	CHL	Children	Stool	2420	84	28 (33.3)	54 (64.3)	–	2 (2.4)
Ghaemi [25]	Iran	2007	Hospital	Children	Stool	634	56	10 (18)	12(22)	3 (5)	31 (55)
Opintan and Newman [12]	Ghana	2007	Hospital	All ages	Stool	594	24	4 (16.7)	17 (70.8)	2 (8.3)	1 (4.2)
Jafari [26]	Iran	2008	Hospital	Children	Stool	1120	157	13 (8)	48 (31)	8 (5)	88 (56)
Orrett [30]	Trinidad	2008	Hospital	All ages	Stool	5187	392	7 (1.8)	75 (19.1)	16 (4.1)	294 (75)
Tiruneh [6]	Ethiopia	2009	Hospital	All ages	Stool	1200	90	9 (10)	65 (72.2)	8 (8.9)	8 (8.9)
Sherchand [15]	Nepal	2009	Hospital	Children	Stool	440	21	14 (66.7)	2 (9.5)	5 (23.8)	–
Zafar [16]	Pakistan	2009	Community	All ages	Stool	8155	394	37 (9)	242 (62)	43 (11)	72 (18)
Pourakbari [17]	Iran	2010	Community	All ages	Stool	15,255	682	34 (5)	327 (48)	14 (2)	307(45)
Xia [27]	China	2011	Hospital	All ages	Stool	3531	467	–	354 (76)	–	113(24)
Nunes [31]	Brazil	2012	Hospital	children	Stool	250	26	–	21 (80.8)	–	5 (19.2)
Khan [28]	Nepal	2013	Hospital	All ages	Stool	507	69	19 (27)	29 (42)	15 (22)	6 (9)
Mengstu [13]	Ethiopia	2014	HC	All ages	Stool	382	17	3 (17.6)	5 (29.4)	3(16.7)	6(35.3)
Mulatu [14]	Ethiopia	2014	Hospital	Children	Stool	158	11	–	11 (100)	–	–
Kumar [29]	India	2014	Hospital	All ages	Stool	1402	146	3 (2.1)	98 (67.1)	8 (5.4)	37 (25.3)
Total						69,849	4027 (5.8)	463 (11.5)	2067 (51.3)	212 (5.3)	1249 (33.2)

A = *Shigella dysentery*, B = *Shigella flexneri*, C = *Shigella boydii*, D = *Shigella sonnei*

*HC* health centre, *PHL* public health laboratories, *CHL* central health laboratories, *Pub* publication, *Pos* positive

*Shigella flexneri* was isolated predominately from Ethiopia [7, 8, 14], Kenya [9, 13], Eritrea [11], Ghana [12], Pakistan [16], Iran [17, 22], China [19, 27], Nepal [23, 28], India [29] and Brazil [31] (Table 1). *S. sonnei* was also isolated predominately from Ethiopia [13], Thailand [18], Vietnam [24] and Iran [25, 26], Chile [21] and Trinidad [30]. *S. dysentery* was also reported majorly from Egypt [10] and Nepal [15]. *S. boydii* did not score highest prevalence in any one of the eligible studies (Table 1).

*Shigella sonnei* was not isolated from studies conducted in Ethiopia [14] and Nepal [20]. *S. dysentery* was not isolated from the studies conducted in Ethiopia [14], Thailand [18], China [19, 27], Chile [21], Brazil [31], and Vietnam [24]. *S. boydii* was not also isolated from the studies conducted in Ethiopia [14], Eritrea [11], Thailand [18], China [19, 27], Chile [21] and Brazil [31] (Table 1).

Nine studies were eligible for antimicrobial susceptibility testing [AST] of *Shigella* Serogroups. Those are three from Africa, five from Asia and one from South America. In all the regions, Ampicillin developed highly resistance to almost all the Serogroups of *Shigella* whereas all the strains were sensitive to Ciprofloxacin. Hundred percent of isolates revealed by Orrette in South America were resistance for Ampicillin but 100 % sensitive to Tetracycline, Chloramphenicol, Ciprofloxacin and Cotrimoxazole (Table 2). In most of the studies observed in Africa (Table 3) and Asia (Table 4), Serogroups of *Shigella* were developed resistance to Tetracycline, Chloramphencol and Cotrimoxazole.

**Table 2:** Review on antimicrobial resistance patterns of Serogroups of *Shigella* conducted in South America

Author	Serogroup	Resistance patterns, N (%)						
		AMP	T	C	CIP	NA	SXT	GM
Orrett [30]	*S. dysentery*	7 (100)	0 (0.0)	0 (0.0)	0 (0.0)	–	0 (0.0)	0 (0.0)
	*S. flexneri*	36 (46)	9 (12)	0 (0.0)	0 (0.0)	–	16 (21)	0 (0.0)
	*S. boydii*	10 (63)	6 (38)	3 (3.9)	0 (0.0)	–	3 (19)	0 (0.0)
	*S. sonnei*	27 (9)	106 (36)	4 (1.4)	2 (0.7)	–	97 (33)	4 (1.4)

**Table 3:** Review on antimicrobial resistance patterns of Serogroups of *Shigella* conducted in Africa

Authors	Serogroups	Resistance patterns, N(%)						
		AMP	T	C	CIP	NA	SXT	GM
Mache [8]	*S. dysentery*	17 (73.9)	15 (64.2)	12 (52.2)	–	11 (8.7)	9 (39.1)	0 (0.0)
	*S. flexneri*	22 (71)	20 (64.5)	13 (41.9)	–	3 (9.7)	9 (29)	1 (3.2)
	*S. boydii*	10 (66.7)	8 (53.3)	4 (25)	–	0 (0.0)	5 (33.3)	0 (0.0)
	*S. sonnei*	5 (62.5)	6 (75)	2 (25)	–	0 (0.0)	2 (25)	0 (0.0)
Tiruneh [6]	*S. dysentery*	8 (89)	7 (77.8)	1 (11)	0 (0.0)	0 (0.0)	7 (77.8)	1 (11)
	*S. flexneri*	54 (83)	62 (95.4)	45 (69)	2 (31)	0 (0.0)	59 (90.8)	7 (10.8)
	*S. boydii*	4 (50)	7 (87.5)	2 (25)	0 (0.0)	0 (0.0)	4 (50)	2 (25)
	*S. sonnei*	3 (37.5)	8 (100)	2 (25)	0 (0.0)	0 (0.0)	6 (75)	1 (12.5)
Naik [11]	*S. dysentery*	19 (95)	–	18 (90)	0 (0.0)	–	0 (0.0)	–
	*S. flexneri*	42 (78)	–	34 (67)	0 (0.0)	–	3 (6)	–

**Table 4:** Review on antimicrobial resistance patterns of Serogroups of *Shigella* conducted in Asia

Author	Serogroups	Resistance patterns, N (%)						
		AMP	T	C	CIP	NA	SXT	GM
Wang [19]	*S. flexneri*	292 (95.4)	–	–	18 (5.9)	305 (99.7)	206 (67.3)	7 (2.3)
	*S. sonnei*	2 (8)	–	–	0 (0.0)	24 (96)	24 (96)	0 (0.0)
Bhattacharya [20]	*S. dysentery*	32 (82.1)	–	–	13 (33.3)	33 (84.6)	35 (89.5)	–
	*S. flexneri*	12 (100)	–	–	2 (16.7)	4 (33.3)	11 (91.7)	–
	*S. boydii*	2 (100)	–	–	1 (50)	0 (0.0)	1 (50)	–
Mashouf [22]	*S. dysentery*	54 (96.4)	50 (89.2)	52 (92.8)	3 (3.5)	48 (85.7)	52 (92.8)	–
	*S. flexneri*	63 (94)	61 (91.1)	61 (91.1)	0 (0.0)	3 (44.7)	59 (88.1)	–
	*S. boydii*	17 (68)	13 (52)	21 (84)	0 (0.0)	0 (0.0)	15 (60)	–
	*S. sonnei*	15 (83.3)	15 (83.3)	17 (94.4)	0 (0.0)	7 (38.8)	14 (77.7)	–
Wilson [23]	*S. dysentery*	6 (75)	7 (87.5)	5 (62.5)	1 (12.5)	5 (62.5)	51 (100)	6 (75)
	*S. flexneri*	33 (64.7)	49 (96)	23 (45.1)	1 (2)	16 (31.4)	8 (100)	34 (66.7)
Jafari [26]	*S. dysentery*	3 (37.5)	8 (100)	0 (0.0)	0 (0.0)	0 (0.0)	7 (87.5)	0 (0.0)
	*S. flexneri*	23 (47.9)	46 (95.8)	23 (47.9)	0 (0.0)	0 (0.0)	42 (87.5)	2 (4)
	*S. boydii*	5 (38.4)	11 (84.6)	1 (7.6)	0 (0.0)	2 (15.4)	10 (76.9)	0 (0.0)

## Discussion

This review addressed the status of the distribution of the Serogroups of *Shigella* and antimicrobial resistance patterns conducted in 25 eligible studies reviewed from Africa, Asia and South America.

*Shigella flexneri* was revealed 100 % from all the eligible studies reviewed in eight African, fourteen Asian and three South American countries. *S. dysentery* was reviewed from 87, 72 and 33 % of the eight African, fourteen Asian and three South American countries respectively. *S. boydii* was reviewed from 75, 79 and 33 % of the studies carried out in eight Africa, fourteen Asia and three South America respectively. *S. sonnei* was also reviewed in 87, 86 and 100 % of the studies conducted in eight Africa, fourteen Asia and three South American countries respectively (Table 1).

Of the total 69,849 stool sample data collected from the 25 eligible studies published from 2001 to 2014, 4027 *Shigella* bacteria were isolated which is 5.8 %. Above 50 % of the proportion of *Shigella* Serogroups was covered by *S. flexneri* which was followed by *S. sonnei* (33 %). *S. boydii* was contributed five percent of the four Serogroups of *Shigella* (Table 1).

The pooled mean resistance of *S. dysentriae* to Ampicillin, Tetracycline, Cotrimoxazole, Chloramphenicol, Nalidix acid, Gentamicin and Ciprofloxacin were 72.1, 69.4, 60, 44, 40, 17 and 7 % respectively. The pooled mean resistance of *S. flexneri to* Tetracycline, Ampicillin, Nalidix acid, Cotrimoxazole, Chloramphenicol, Gentamicin and Ciprofloxacin were 75.8, 75.6, 74.5, 72.7, 51.7, 14.5 and 7 % respectively. The pooled mean resistance of *S. boydii* to Ampicillin, Tetracycline, Cotrimoxazole, Chloramphenicol, Ciprofloxacin, Gentamicin and Nalidix acid were 64, 63, 48, 29, 10, 6 and 3 % respectively. The pooled mean resistance of *S. sonnei* to Tetracycline, Cotrimoxazole, Nalidix acid, Ampicillin, Chloramphenicol, Gentamicin and Ciprofloxacin were 79, 71, 54, 47, 35, 16 and 0 % respectively (Table 5).

**Table 5:** Pooled range and mean of antimicrobial resistance patterns of *Shigella* Serogroups

Serogroups of *Shigella*	Resistance (%)													
	AMP		T		C		CIP		NA		SXT		GM	
	PR	PM	PR	PM	PR	PM	PR	PM	PR	PM	PR	PM	PR	PM
*S. dysentery*	37.5−100	72.1	0−100	69.4	0−92.8	44.1	0−33.3	7	0−85.7	40.3	0−100	60.8	0−75	17.2
No. of studies	8		6		7		7		6		8		5	
*S. flexneri*	46−100	75.6	12−95.8	75.8	0−91.1	51.7	0−31	7	0−99.7	74.1	6−100	72.7	0−66.7	14.5
No. of studies	9		6		7		8		7		9		6	
*S. boydii*	38.4−100	64.4	38−87.5	63.1	3.9−84	29.1	0−50	10	0−15.4	3.1	19−76.9	48.2	0−25	6.3
No. of studies	6		5		5		5		5		6		4	
*S. sonnei*	8−83.3	46.9	36−100	79.4	1.4−94.4	35.4	0−0.7	0.12	0−96	54.8	25−100	71.9	0−12.5	16.6
No. of studies	7		6		6		6		6		6		6	

*AMP* Ampicillin, *T* Tetracycline, *C* Chloramphenicol, *CIP* Ciprofloxacin, *NA* Nalidixic acid, *SXT* sulphamethoxazole trimethoprim/Cotrimoxazole, *GM* gentamicin, *PR* pooled range, *PM* pooled mean, *No* Number

Above 50 % of all Serogroups of *Shigella* developed resistance to Ampicillin, Tetracycline, Cotrimoxazole and Chloramphenicol which are the commonly prescribed antimicrobial drugs.

## Conclusions

The incidence of *Shigella* Serogroups in the selected three regions is different. The domination of *S. flexneri* is observed in Africa and Asia although *S. sonnei,* the most dominant in South America, is predominately isolated in one study in Ethiopia. This may give clue to the scientific world about the migration and movement of strains from one region to the other region. *Shigella* Serogroups are becoming resistance to the commonly prescribed antimicrobial drugs in developing countries.
